# Saturated genic SNP mapping identified functional candidates and selection tools for the *Pinus monticola Cr2* locus controlling resistance to white pine blister rust

**DOI:** 10.1111/pbi.12705

**Published:** 2017-03-17

**Authors:** Jun‐Jun Liu, Richard A. Sniezko, Arezoo Zamany, Holly Williams, Ning Wang, Angelia Kegley, Douglas P. Savin, Hao Chen, Rona N. Sturrock

**Affiliations:** ^1^ Canadian Forest Service Natural Resources Canada Victoria Canada; ^2^ Dorena Genetic Resource Center USDA Forest Service Cottage Grove OR USA; ^3^ Academy of Agriculture and Forestry Science Qinghai University Xining China

**Keywords:** candidate gene approach, genetic map, marker‐assisted selection, SNP genotyping, white pine blister rust

## Abstract

Molecular breeding incorporates efficient tools to increase rust resistance in five‐needle pines. Susceptibility of native five‐needle pines to white pine blister rust (WPBR), caused by the non‐native invasive fungus *Cronartium ribicola* (J.C. Fisch.), has significantly reduced wild populations of these conifers in North America. Major resistance (R) genes against specific avirulent pathotypes have been found in several five‐needle pine species. In this study, we screened genic SNP markers by comparative transcriptome and genetic association analyses and constructed saturated linkage maps for the western white pine (*Pinus monticola*) R locus (*Cr2*). Phenotypic segregation was measured by a hypersensitive reaction (HR)‐like response on the needles and disease symptoms of cankered stems post inoculation by the *C. ribicola avcr2* race. SNP genotypes were determined by HRM‐ and TaqMan‐based SNP genotyping. Saturated maps of the *Cr2*‐linkage group (LG) were constructed in three seed families using a total of 34 SNP markers within 21 unique genes. *Cr2* was consistently flanked by contig_2142 (encoding a ruvb‐like protein) and contig_3772 (encoding a delta‐fatty acid desaturase) across the three seed families. *Cr2* was anchored to the *Pinus* consensus LG‐1, which differs from LGs where other R loci of *Pinus* species were mapped. GO annotation identified a set of NBS‐LRR and other resistance‐related genes as R candidates in the *Cr2* region. Association of one nonsynonymous SNP locus of an NBS‐LRR gene with *Cr2*‐mediated phenotypes provides a valuable tool for marker‐assisted selection (MAS), which will shorten the breeding cycle of resistance screening and aid in the restoration of WPBR‐disturbed forest ecosystems.

## Introduction

Western white pine (*Pinus monticola* Douglas ex D. Don) is highly susceptible to white pine blister rust (WPBR). This rust disease is caused by an exotic fungal pathogen *Cronartium ribicola* J.C. Fisch., which was introduced into North America around 1910. Since then, *C. ribicola* has spread to most regions where native five‐needle pines (subgenus *Strobus*) are naturally distributed. Due to a variety of factors, including high susceptibility and mortality to WPBR, the frequency of *P. monticola* in natural stands has been reduced by up to 95% (Fins *et al*., 2002). Management practices for control of WPBR include cleaning and disinfection of nurseries, eradication of *Ribes* (alternate host plants), developing rust hazard zones, pruning infected branches and breeding for host resistance (Geils *et al*., 2010). Breeding programmes have identified major resistance (R) loci (*Cr1* to *Cr4*) in four five‐needle species: sugar pine (*P. lambertiana* Doug.), western white pine, south‐western white pine (*P. strobiformis* Engelm.) and limber pine (*P. flexilis* James) (Kinloch and Dupper, 2002; Kinloch *et al*., 1970, 1999; Schoettle *et al*., 2014; Sniezko *et al*., 2008, 2016). These R loci are characterized by Mendelian inherited resistance traits usually featured as an absence of stem cankers following inoculation by the relevant avirulent rust isolates. In addition, quantitative traits for partial resistance against WPBR have been observed and are proposed to be more durable to a wide spectrum of *C. ribicola* races (Sniezko *et al*., 2008, 2014).

Pyramiding resistant alleles of different R loci has been demonstrated to be an effective breeding strategy to confer much stronger and environmentally stable resistance in agricultural crops (Fukuoka *et al*., 2015). Because quantitative trait loci (QTL) are subject to lower selection pressures than major gene resistance (MGR) conferred by a single dominant R gene, a promising approach to sustain long‐term resistance to WPBR is the incorporation of partial resistance mechanisms (or QTLs) and multiple R genes (Kinloch *et al*., 2012; Sniezko *et al*., 2014). Several genes with minor effects contributing to partial resistance to WPBR have been identified (Liu *et al*., 2011, 2013a,c, 2016b), but putative QTLs still await genetic characterization in all of the WPBR pathosystems. Towards realizing this ideal strategy to control WPBR, we need to understand the genetic relationships among R genes and among QTLs. Therefore, molecular characterization of R loci and development of marker‐assisted selection (MAS) or other genomics‐based diagnostic tools are important strategies used to improve breeding practices (Sniezko *et al*., 2014; Yang *et al*., 2015).

Genetic mapping of linkage groups (LG) with DNA markers at high density is commonly used as one of the most valuable research approaches to address the need for high‐throughput selection of superior plant traits of economical or ecological interest among various germplasms. Molecular mapping of plant R genes has been used to dissect the genetic architecture of host resistance, providing an effective research strategy to develop diagnostic markers in plant breeding (Miedaner and Korzun, 2012). In *P. monticola*, the *Cr2* locus confers complete resistance to the *C. ribicola avcr2* race and is characterized phenotypically by a hypersensitive reaction (HR)‐like defence response on needles post basidiospore infection (Kinloch and Dupper, 2002). *Cr2* was mapped previously using markers of random amplified polymorphic DNA (RAPD), amplified fragment length polymorphism (AFLP), sequence‐characterized amplified region (SCAR) and resistance gene analogs (RGA)‐based AFLP (Liu and Ekramoddoullah, 2008). However, genetic maps with nongenic DNA markers have limited future application due to a low transferability between seed families, making development and application of MAS tools difficult in a wide range of germplasms. For comparative mapping of R loci among *Pinus* species, it is necessary to develop genic markers with sufficient sequence similarity among related protein‐coding regions.

Compared to traditional DNA markers, single nucleotide polymorphisms (SNPs), which can be detected across the whole genome by next‐generation sequencing (NGS), provide the richest resource for detection of genic variations. A large number of SNP and satellite markers were discovered in *P. monticola* by RNA‐seq‐based transcriptome profiling (Liu and Hammett, 2014; Liu *et al*., 2013b) and high‐throughput SNP genotyping identified a set of genic markers that showed linkage disequilibrium (LD) with *Cr2* in a collection of breeding germplasms (Liu *et al*., 2014). Comparative SNP genotyping positioned *P. lambertiana Cr1* against *C. ribicola avcr1* race on the *Pinus* consensus LG‐2 (Jermstad *et al*., 2011; Neale *et al*., 2014) and *P. flexilis Cr4* against *C. ribicola avcr4* on the *Pinus* consensus LG‐8 (Liu *et al*., 2016a). The *P. taeda* R gene *Fr1* against the rust fungus *C. quercuum* (f.sp. *fusiforme*) was mapped to LG‐2 (Neale *et al*., 2014; Quesada *et al*., 2013). These genetic maps of *Pinus* R genes provide valuable genetic and genomic resources for further development of high‐density genetic maps aimed towards the positional cloning of conifer R genes.

Genomic approaches have shown great potential for enhancing plant breeding (Neale *et al*., 2013). Availability of genome sequences of *P. taeda* (v1.1) and *P. lambertiana* (v1.0) through the PineRefSeq project (Gonzalez‐Ibeas *et al*., 2016; Stevens *et al*., 2016; Wegrzyn *et al*., 2014) allows improved predictions of gene conservation in other *Pinus* genomes. Transcriptome profiling revealed defence‐responsive genes and RGAs of the nucleotide‐binding site‐leucine‐rich repeat (NBS‐LRR) and receptor‐like protein kinase (RLK) families during white pine blister rust interactions (Liu *et al*., 2013b, 2014), providing candidates to explore gene variations and functional contributions to host resistance against WPBR. Recent progress in genome‐wide gene annotation, transcript expression and gene variation profiles has accumulated genomic resources for comprehensive understanding of tree genetic resistance mechanisms, enabling elucidation of R gene evolution and development of diagnostic markers for MAS in forest tree breeding (Plomion *et al*., 2016; Sniezko *et al*., 2014). Application of MAS tools for prediction and diagnosis of pest/pathogen resistance can help plant breeders to reduce breeding cycle times and the costs associated with selection of desired phenotypes. Development and application of diagnostic markers in five‐needle pine breeding and conservation programmes holds high potential to expedite restoration of ecosystems damaged by WPBR.

**Figure 1:**
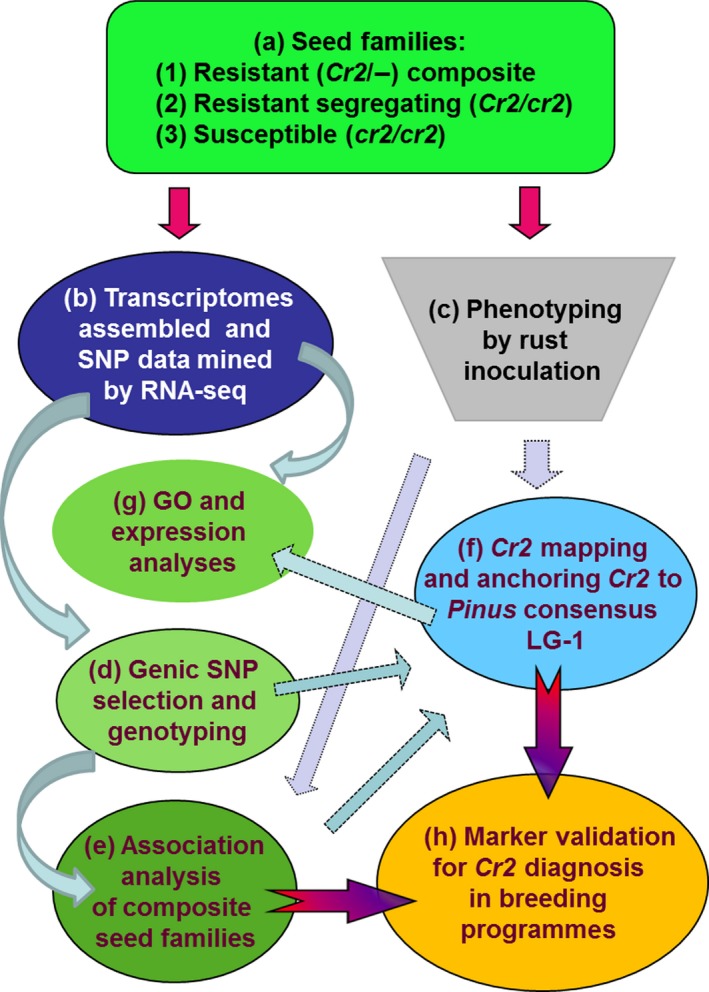
Research steps towards the identification of *Cr2* candidates and development of diagnostic markers for *Cr2* selection using an integrated genomics strategy. (a) Three types of seed families were used in the present study: resistant segregating (*Cr2/cr2*), susceptible (*cr2/cr2*) and resistant composite (*Cr2/−*) seed families. (b) Transcriptomes assembled and SNP data mined by RNA‐seq analysis were available from previous reports (Liu *et al*., 2013b, 2014), provided candidate genes and *in silico* SNP data set for step (d). (c) Phenotyping by rust inoculation provided samples and phenotypic data of the resistant (*Cr2/−*) composite seed families for association analysis at step (e); it also provided samples and phenotypic data of resistant segregating (*Cr2/cr2*) seed families for step (f). (d) SNP loci were selected from candidate genes and by comparison of transcriptomes between resistant (*Cr2/−*) and susceptible (*cr2/cr2*) trees or between *P. monticola* and *P. taeda*. SNP genotyping was performed using HRM and TaqMan technologies. (e) Association analysis was performed based on resistance‐related phenotypic and SNP genotypic data from composite seed families. (f) *Cr2* mapping was performed using three resistant segregating (*Cr2/cr2*) seed families and it localized genes in the *Cr2* region and anchored *Cr2* on the *Pinus* consensus LG‐1. (g) The mapped genes were subjected to gene ontology (GO) analysis by BLAST2GO and expression analysis using RNA‐seq data. (h) Marker validation for *Cr2* diagnosis in breeding programmes was performed by TaqMan genotyping arrays.

To develop MAS tools for *Cr2* selection and to facilitate *Cr2* characterization by positional cloning and functional candidate analysis, we report here on the saturated genetic mapping of *Cr2* using genic SNP loci and identification of putative functional candidates at the *Cr2* locus, using an integrated genomics strategy (Figure 1). High‐resolution melting (HRM) analysis‐ and TaqMan array‐based SNP genotyping mapped 21 *P. monticola* genes on the *Cr2*‐LG using haploid megagametophyte populations of three seed families. Comparative analysis revealed a general colinearity between the *Cr2* region and the orthologous genomic regions of the *Pinus* consensus LG‐1. Linkage analysis consistently mapped *Cr2* between SNP markers snp2142‐980Y and snp3772‐739R, across the three seed families. Gene ontology (GO) analysis and gene annotation demonstrated that the *Cr2* region contains multiple genes with putative functions in disease resistance, including those encoding NBS‐LRR proteins, F‐box proteins and fatty acid desaturase.

## Results

### Genotype–phenotype (G × P) association using SNP genotypic and *Cr2*‐mediated phenotypic data

Seedlings of resistant composite (*Cr2/−*) seed families and resistant segregating (*Cr2/cr2*) seed families (#3566, #3592 and #1974) were evaluated for disease resistance phenotypes after *C. ribicola* inoculation (Figure 1a, c). A set of 432 SNP loci were previously genotyped in 376 seedlings of the resistant composite (*Cr2/−*) seed families (Liu *et al*., 2014). Based on phenotypic and genotypic data from these resistant composite (*Cr2*/*−*) seed families, G × P association analysis (Figure 1e) revealed a total of 12 SNP loci in significant association with the susceptible phenotype (Table 1). *R* ^2^ for these 12 SNP markers ranged from 0.1 to 0.9 (*P *< 0.01 after correction by 10^5^ permutations). Among all tested SNP loci, snp41490‐1778M showed the strongest association with *cr2*‐mediated susceptibility (*R* ^2^ = 0.9). The 12 SNP loci showing phenotypic association were distributed in eight functional genes: one gene with three SNP loci (contig_3772), two genes each with two SNP loci (contig_41490 and contig_11322) and five other genes each with one SNP locus (Table 1).

**Table 1:** Association analysis of SNP genotypic data with phenotypic traits (*Cr2*‐mediated resistance) using TASSEL

Trait	Gene ID	SNP marker ID	marker_p^a^	perm_p^b^	marker_ Rsq^c^	marker _df^d^	error df^e^	Mapped
SUS	Putative cr2	Putative *cr2/cr2*	0	1.00E−05	1	1	277	Yes
SUS	contig_41490	snp41490‐1778M	6.57E−60	1.00E−05	0.90071	2	118	Yes
SUS	contig‐4105	snp4105‐1000S	1.04E−63	1.00E−05	0.80936	2	175	Yes
SUS	contig_41490	snp41490‐1618R	1.30E−38	1.00E−05	0.65938	2	162	Yes
SUS	contig_11322	snp11322‐121R	3.87E−33	1.00E−05	0.57802	2	173	Yes
SUS	contig_1471	snp1471‐201S	5.02E−31	1.00E−05	0.54947	2	175	Yes
SUS	contig_573	snp573‐186Y	2.22E−24	1.00E−05	0.47511	2	169	Yes
SUS	contig_3772	snp3772‐739R	3.24E−23	1.00E−05	0.44669	2	175	Yes
SUS	contig_11322	snp11322‐190K	3.86E−21	1.00E−05	0.40172	2	183	No
SUS	contig_3772	snp3772‐1107Y	2.04E−19	1.00E−05	0.37845	2	181	Yes
SUS	contig_3772	snp3772‐858Y	2.19E−19	1.00E−05	0.37154	2	185	No
SUS	contig_3186	snp3186‐356W	2.09E−06	5.60E−04	0.15083	2	160	No
SUS	contig_3704	snp3704‐190S	2.95E−05	0.00627	0.10887	2	181	No

a marker_P: *P* value from the *F*‐test on the SNP marker.

b perm_p: *P* value from the *F*‐test on the marker after correction by 100 000 permutations.

c marker_Rsq: *R* ^2^ for the marker after fitting other model terms (population structure).

d marker_df: Degree freedom of marker.

e error_df: Degree freedom of residual error.

### SNP genotyping

**Figure 2:**
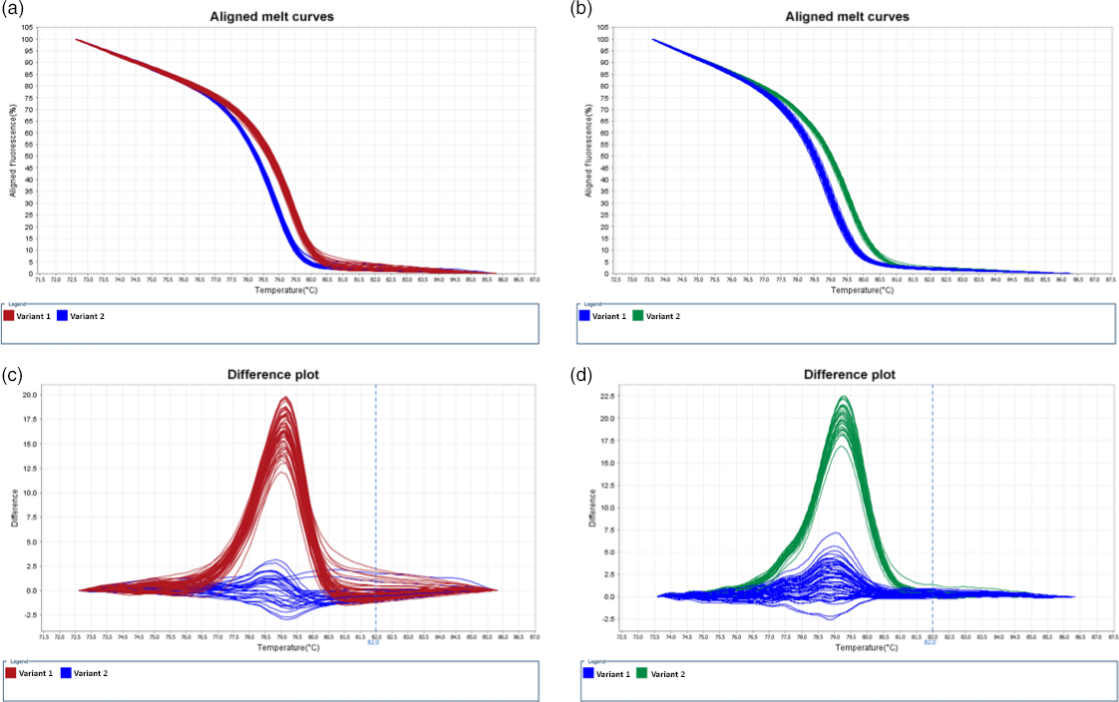
Genotyping of single nucleotide polymorphisms (SNP) using high‐resolution melt (HRM) analysis. PCR fragments were amplified from the targeted *Pinus monticola* genes. (a) Aligned melt curves for snp11322‐121R in seed family #3566. (b) Aligned melt curves for snp573‐186R in seed family #1974. (c) Difference plot for snp11322‐121R in seed family #3566. (d) Difference plot for snp573‐186R in seed family #1974.

SNP loci of these eight genes in significant association with the susceptible phenotype (Table 1) were first selected to verify potential genetic linkage of their alleles to *Cr2* in resistant, segregating (*Cr2/cr2*) seed families by haploid segregation analysis. SNP genotypes of each individual megagametophyte sample were determined by the HRM‐based SNP genotyping technology (Figure 2). Following chi‐square tests of SNP genotypic data, six genes (Table 2) showed an allelic segregation (1:1 ratio) in at least one of three mapping seed families. The other two genes identified in the association study (contig_3186 and contig_3704) had monomorphic sites and their linkage to *Cr2* was not subjected to further confirmation in this study.

**Table 2:** BLASTn search of *21 Pinus monticola Cr2*‐linked genes against *P. taeda* genes mapped on the consensus linkage groups

Gene ID^a^	Lowest *E*‐value^b^	Pita gene ID^c^	Sequence description^d^	Mapped SNP loci (ID)	Mapped SNP loci (n)
contig_4105	0.00E+00	0_18435 (1363356)	f‐box kelch repeat protein	snp4105‐24R, snp4105‐1000S	2
contig_1471	1.65E−82	CL199Contig3	Plasma membrane associated protein	snp1471‐201S	1
contig_13194	0.00E+00	0_17655 (1362576)	Bromodomain‐containing protein 9	snp13194‐1071Y, snp13194‐1820R	2
contig_9279	0.00E+00	2_2559 (1354522)	atp synthase subunit b chloroplastic	snp9279‐576Y	1
contig_34906	0.00E+00	ContigId:Contig5830	Probable alanine–trna chloroplastic isoform x1	snp34906‐864K	1
contig_8821	0.00E+00	ContigId:Contig5113	Peptidase m23	snp8821‐877Y	1
contig_12508	0.00E+00	2_5726 (1371316)	o‐glucosyltransferase rumi homolog	snp12508‐611Y, snp12508‐1460R	2
contig_2142	0.00E+00	2_9847 (1375437)	ruvb‐like 2	snp2142‐980Y, snp2142‐1542K	2
contig_186209	0.00E+00	0_14523 (1359444)	Uncharacterized loc101202700	snp186209‐285M, snp186209‐573Y, snp186209‐681R	3
contig_7093	0.00E+00	ContigId:Contig828	Trichoplein keratin filament‐binding isoform x4	snp7093‐883K	1
contig_2711	0.00E+00	ContigId:Contig1913	Methylthioribose kinase	snp2711‐366Y	1
contig_33357	0.00E+00	2_3136 (1376615)	DnaJ/HSP40	snp33357‐554S	1
contig_29619	0.00E+00	0_2366 (1353774)	Uridine‐cytidine kinase c isoform x1	snp29619‐877Y, snp29619‐2150R	2
contig_41490	3.41E−18	0_848 (1376507)	Truncated tir‐nbs‐lrr protein	snp41490‐654Y, snp41490‐1618R, snp41490‐1778M, snp41490‐2928R, snp41490‐3491Y	5
contig_58688	6.08E−17	0_4134 (1382924)	nbs‐lrr protein	snp58688‐438M	1
contig_176313	1.22E−11	0_335 (1377266)	nbs‐lrr protein	snp176313‐250K	1
contig_189504	8.33E−05	0_4134 (1382924)	nbs‐lrr protein	snp189504‐477R	1
contig_13287	4.06E−02	0_9466 (1365041)	Proline‐rich protein	snp13287‐508R, snp13287‐956Y	2
contig_573	8.60E−02	0_13169 (1358090)	Pyruvate dehydrogenase (acetyl‐transferring) mitochondrial	snp573‐186Y	1
contig_11322	9.88E−02	0_9001 (1364576)	f‐box lrr‐repeat protein 14	snp11322‐121R	1
contig_3772	2.38E−01	0_16998 (1361919)	Delta ‐fatty‐acid desaturase‐like	snp3772‐739R, snp3772‐1107Y	2

a Six genes were selected by association analysis, and other 15 genes were selected by comparative transcriptome analysis.

b *E*‐values as indicators for gene homology levels between pine species were calculated by BLASTn using CLC Genomics Workbench (v.5.5).

c *Pinus taeda (Pita)* gene identifications were based on Westbrook *et al*. (2015).

d Putative functions of western white pine genes were predicted by a BLASTx search against the NCBI nr database using BLAST2GO.

To enrich the *Cr2* region with additional SNP markers, *in silico* SNP loci, discovered previously by transcriptome sequencing (Figure 1b; Liu *et al*., 2013b, 2014), were screened in the highly conserved *Pinus* genes and resistance‐related candidate genes. SNP screening was based on transcriptome comparisons between *P. monticola* resistant (*Cr2/cr2*) and susceptible (*cr2/cr2*) seed families, as well as between *P. monticola* transcripts and *P. taeda* genomic contigs (Figure 1d). Among genes that were polymorphic only in resistant trees (Liu *et al*., 2014), 15 were selected for SNP genotyping in resistant, segregating (*Cr2/cr2*) seed families used for *Cr2* mapping. In total, 21 functional genes were genotyped by HRM‐based analysis (Figure 1d). At least two SNP loci were consistently genotyped in nine genes, with a total of 34 SNP loci analysed in 21 genes (Table 2, Table S1). Genotypic data of these 34 SNP loci within 21 genes were successfully collected in 128, 80 and 287 megagametophyte samples in three resistant, segregating (*Cr2/cr2*) seed families (#3566, #3592 and #1974, respectively) and used to construct genetic maps.

To further confirm genotypes of SNP loci, Sanger sequencing was performed on PCR fragments encompassing SNP loci from genomic DNA of haploid tissues (Figure S1). Alignment analysis of DNA sequences demonstrated a complete concordance to the genotypes assigned by SNP genotyping methods, providing independent and direct evidence for the accuracy of *in silico* SNPs detected by RNA‐seq analysis, the Sequenom and HRM genotyping methods.

### Saturated *Cr2* genetic maps

**Figure 3:**
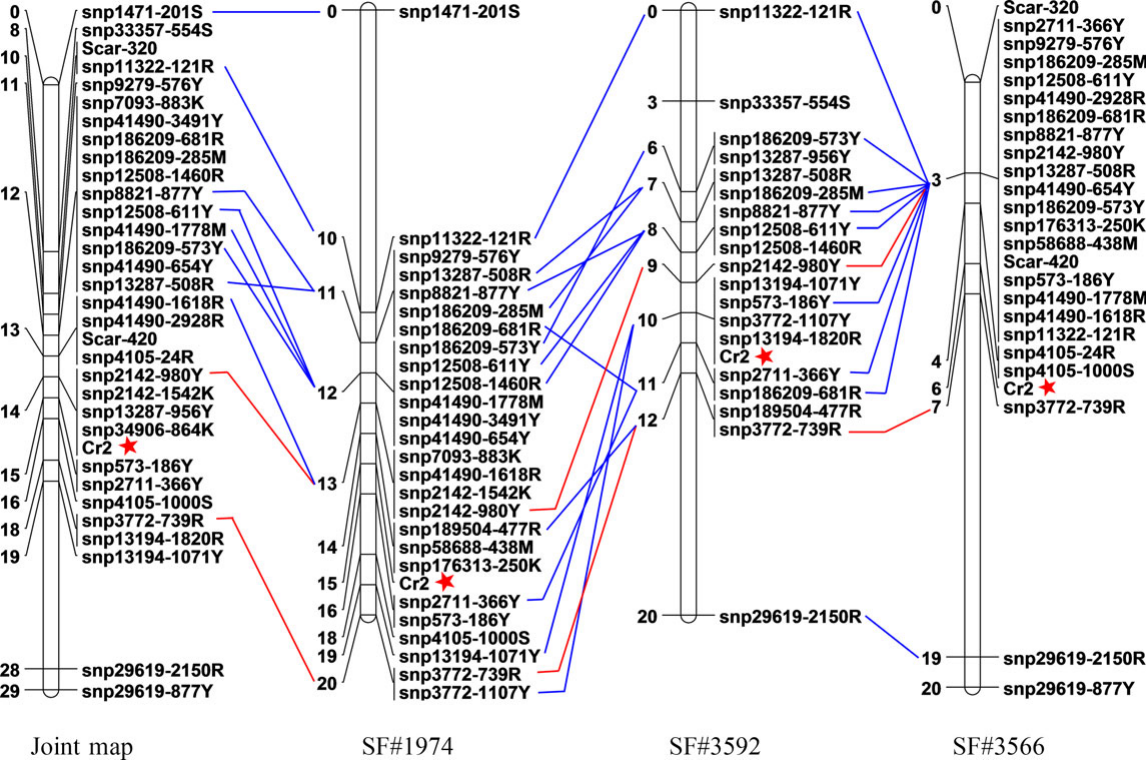
Genetic linkage maps of *Pinus monticola Cr2* using SNP markers in seed families #3566, #3592 and #1974. Two SNP markers, snp2142‐980Y and snp3772‐739R, flanking *Cr2* consistently across three seed families are shown by red lines. Two SCAR markers from previous work are included in the seed family #3566.

Using JoinMap software, linkage analysis mapped 19, 22 and 26 SNP markers on the *Cr2*‐LG in the seed families #3592 (LOD ≥9), #3566 (LOD ≥10) and #1974 (LOD ≥10), respectively. The *Cr2*‐LG lengths were about 20 cM in all three seed families, with a mean locus density of <1 cM per SNP marker (Figure 3). *Cr2* was localized between two flanking SNP loci snp2142‐980Y and snp3772‐739R, which was consistent in the three seed families. Genetic distance between two consistent flanking SNP markers was measured at ~4.5 cM on an integrated map combined from three populations, and *Cr2* was mapped at distances of 0.9 cM proximal to snp2142‐980Y and 3.6 cM distal to snp3772‐739R. A total of 21 SNP markers within 14 genes were positioned in this *Cr2* region in at least one seed family (Figure 3).

### *Cr2* was anchored on the *Pinus* consensus LG‐1

To reveal genomic content within the *Cr2* region, sequences of 21 *Cr2*‐linked genes were used to identify the corresponding regions of the *P. taeda* genome. BLASTn analysis against the *P. taeda* genome draft (Zimin *et al*., 2014) showed that all 21 *Cr2*‐linked genes were highly conserved between the two *Pinus* species. Compared to *P. taeda* mapped sequences (Eckert *et al*., 2009; Neves *et al*., 2014; Westbrook *et al*., 2015), 13 *Cr2*‐linked genes showed significant homologies between the two *Pinus* species: 12 with identical hits (BLASTn *E*‐value = 0) and one with a highly homologous hit (1.651E−82) (Table 2). All 13 *P. taeda* genes were localized on LG‐1 of the *Pinus* consensus maps (Westbrook *et al*., 2015), and nine of them were assigned a position on the *Pinus* consensus LG‐1 (Figure S2).

**Figure 4:**
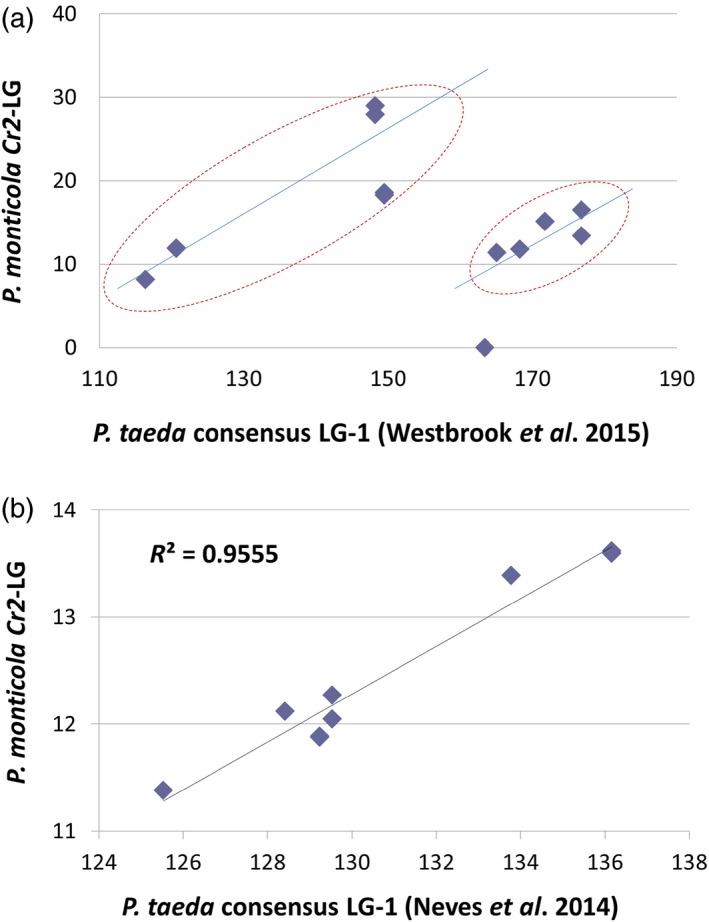
Syntenic relationship between *Pinus monticola* Cr2‐linkage group and *P. taeda* consensus LG‐1. A dot plotting was performed using map position data of the *Pinus* highly conserved genes mapped in both species. *P. taeda* genetic map data were based on two previous reports: (a) Westbrook *et al*. (2015), (b) Neves *et al*. (2014).

Correlation analysis of the gene order and positions revealed a linear relationship with two discontinued segments between *Cr2*‐LG and the *Pinus* consensus LG‐1 (Figure 4a). As evaluated by the Pearson correlation coefficient, two segments showed high consistencies between the two *Pinus* species: the proximal segment containing seven SNP loci from four unigenes with *R* = 0.84 (*P* = 0.017), and the distal segment containing five SNP loci, from four unigene, with *R* = 0.77 (*P* = 0.12). When *Cr2*‐LG was compared with the *P. taeda* genetic map developed by Neves *et al*. (2014), the distal segment showed a better correlation with *R* = 0.97 (*P *<* *0.00001), where nine SNP loci from six unigenes were covered (Figure 4b). These results indicate that *Cr2* is anchored on the *Pinus* consensus LG‐1.

### Gene annotation identified NBS‐LRR and other disease resistance‐related genes at the *Cr2* locus

To determine R candidates in the *Cr2* region, GO analysis was used to annotate genes mapped on the *Cr2*‐LGs (Table 2; Figure 1g). Of 13 genes localized in the *Cr2* region between SNP markers snp2142‐980Y and snp3772‐739R, four genes (contig_176313, contig_58688, contig_189504 and contig_41490) encoded putative NBS‐LRR proteins. Among well‐characterized plant R proteins, contig_41490 showed the highest homology to the tobacco TMV R protein N (U15605) by BLASTp analysis with an *E* value of 2e−86. Sequence alignment using Clustal Omega revealed 29.4% amino acid identity across the full‐length sequences between these two proteins (Figure S3). Alignment analysis of Sanger sequences showed contig_176313 and contig_58688 shared 90% overall identity and belong to the same gene cluster. Nucleotide identities of the NBS‐coding regions ranged from 61.5% (contig_41490 vs. contig_176313/contig_58688) to 69.8% (contig_41490 vs. contig_189504).

Seven other genes may contribute to host disease resistance, including two encoding F‐box proteins (contig_4105 for F‐box with C‐terminal kelch repeats, FBK; and contig_11322 for F‐box with C‐terminal LRR domains, F‐LRR), contig_2142 encoding a ruvb‐like protein, contig_13194 encoding bromodomain‐containing protein, contig_13287 encoding a proline‐rich protein, contig_573 encoding mitochondrial pyruvate dehydrogenase (PDH) and contig_3772 encoding delta‐fatty acid desaturase‐like protein. Genes homologous to these have previously been reported to be involved in the plant defence response against pathogen attacks or to be a component in plant immune systems.

Three other genes were mapped within the *Cr2* region: contig_2711 (a homolog of methylthioribose kinase), contig_34906 (a homolog of alanine–tRNA ligase, chloroplastic isoform) and contig_186209 (a tyrosine sulfotransferase‐like). These genes have not yet been reported to be involved in disease resistance. Our study did not detect a significant change in transcript levels for these three genes during white pine blister rust interaction, suggesting that they may not directly contribute to disease resistance against *C. ribicola*. Identification of NBS‐LRR genes and other disease resistance‐related genes in the *Cr2* region could be valuable in breeding new rust‐resistant seed families and gaining a deeper understanding of disease response mechanisms in western white pine.

### Transcript expression of the functional candidates in the *Cr2* region

**Figure 5:**
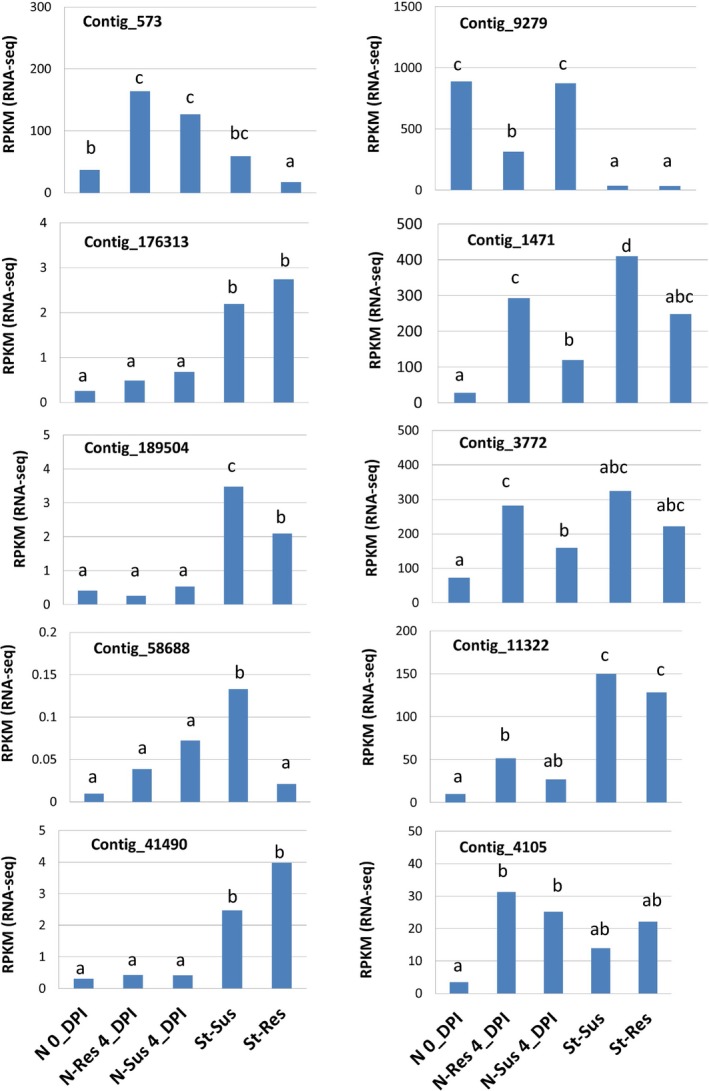
Transcript expression profiles of *Pinus monticola Cr2*‐linked genes by RNA‐seq analysis. Gene expression levels were evaluated by RPKM using the CLC program. Needles and stems were sampled from 6‐month‐old and 2‐year‐old seedlings, respectively. N 0_DPI: needles were sampled to analyse regulation of transcripts in response to infection by *Cronartium ribicola* at 0 day post infection (0_dpi); N‐Res 4_DPI: needles of resistant (*Cr2/−*) seedlings were sampled at 4 days post infection; N‐Sus 4_DPI: needles of susceptible (*cr2/cr2*) seedlings were sampled at 4 days post infection; St‐Res: healthy stem tissues were sampled from resistant (*Cr2/−*) seedlings (cankered‐free); and St‐Sus: cankered stems of susceptible (*cr2/cr2*) seedlings) were samples at 13 months post infection. Different letters indicate significant differences among sample sets (Kal's test and *t*‐test, *P* < 0.05 corrected by the false discovery rate‐FDR).

Because mapped SNP loci were selected by a comparative transcriptomics analysis between resistant and susceptible trees, all mapped genes were expressed as expected, thus indicating their active transcription in needle and stem tissues. Six genes (contig_11322, F‐LRR; contig_3772, delta‐fatty acid desaturase‐like; contig_4105, FBK; contig_573, mitochondrial PDH; contig_1471, plasma membrane‐associated protein; and contig_9279, chloroplastic ATP synthase subunit b) showed significant regulation of their mRNA expression in the primary needle tissues after *C. ribicola* inoculation. All of these genes were up‐regulated except for contig_9279 which was down‐regulated in resistant seedlings (Figure 5). Transcripts of four genes (contig_189504, contig_58688, contig_573 and contig_1471) were measured at levels significantly higher in the susceptible cankered stem tissues than in the resistant non‐cankered stem tissues (Figure 5), indicating a defence‐responsive expression in stem tissues.

All four NBS‐LRR genes showed significantly higher levels of transcript expression in stem tissues of 2‐year‐old seedlings than in the primary needles of 6‐month‐old seedling, and two genes (contig_189504 and contig_58688) showed defence‐responsive expression in stem tissues during *C. ribicola* infection (Figure 5). A large proportion of *Cr2*‐linked genes were up‐regulated due to WPBR infection, which suggests a coordinated regulation among these components in disease resistance.

### Development and validation of a TaqMan tool for *Cr2* selection

**Figure 6:**
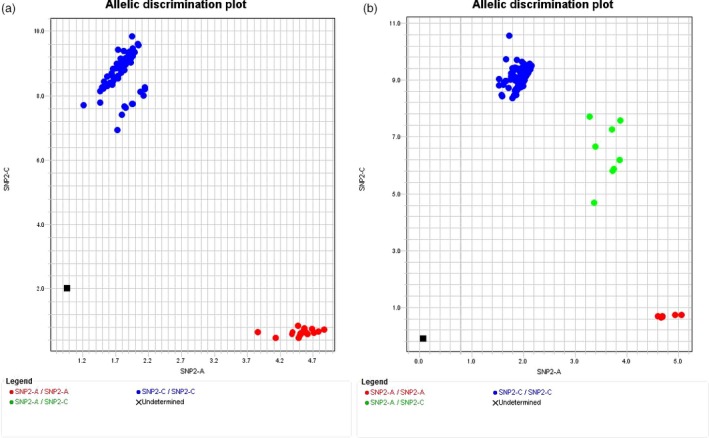
Genotyping of single nucleotide polymorphisms (SNP) using TaqMan technology. Allelic discrimination of SNP genotypes was plotted using TaqMan assay snp41490‐1778M among individual samples. Genomic DNA was extracted from megagametophyte tissues (a), or needle tissues (b). The genotypes (A/A) and (A/C) with linkage to resistance are in red and green, and the genotype (C/C) with linkage to susceptibility is in blue, respectively.

Two SNP loci (snp41490‐1618R and snp41490‐1778M) were used to develop TaqMan SNP genotyping arrays (Figure 6, Table S2). TaqMan genotyping results confirmed all sample genotypes as detected in haploid megagametophyte tissues by HRM analysis in this study, as well as in diploid needle tissues as previously detected by Sequenom technology (Liu *et al*., 2014).

To validate the usefulness of TaqMan tools in breeding programmes, the TaqMan array snp41490‐1778M was used to genotype *Cr2*‐resistant parental trees used in western white pine breeding programmes in Oregon (OR) and British Columbia (BC) as well as seed families with partial resistance (bark reaction) (Table 3, S3). Among these genotyped samples, the composite‐resistant families (*Cr2/−*) were detected as a mixture from approximately 71 full‐sib families (Liu *et al*., 2014). The susceptible (*cr2/cr2*) seed families represented a wide geographical distribution in western North America (Table S3). Genotyping results for samples having phenotypic data confirmed a 95% of G × P match rate (Table 3), similar to that detected by the association study using genotypic data from Sequenom SNP genotyping technology (Table 1).

**Table 3:** Genotype and phenotype (G × P) match rate of western white pine seed families used in breeding programmes

Seed family ID	Genotypes of snp41490‐1778M			Subtotal	Phenotype*	G × P match rate
	A/A	A/C	C/C			
Composite seed families (*Cr2/−*)	93	159	7	259	Res	0.97
	2	13	235	250	Sus	0.94
OR Res parental trees (Cr2/*−*)	2	16	1	19	Res	0.95
BC Res parental trees (Cr2/*−* and *cr2/cr2*)	2	25	13	40	Res or Sus	na
(BC_#3671) X Wind (W)			15	15	Sus	1
(BC_#3674) X W		2	13	15	Sus	0.87
Seed families (*cr2/cr2*) with bark reaction (SY2003)						
(03014‐150 x w) X (03015‐022 x 03013‐002)			11	11	Sus	
(06025‐532 x w) x (06025‐504)			12	12	Sus	
(05014‐124 x w) x (05015‐030 x 03013‐015)			9	9	Sus	
(06024‐506) X (06024‐504 x 511)			9	9	Sus	
(18034‐389) X (18034‐380)			11	11	Sus	
(21105‐052) X W			8	8	Sus	
(05082‐413) X W			10	10	Sus	
(05081‐003) X W			11	11	Sus	
(03024‐783) X W			4	4	Sus	
(03024‐784) X W		2	5	7	Sus	
(03024‐856) X W		2	5	7	Sus	
(06125‐568) X W		1	3	4	Sus	
(06025‐308) X W			4	4	Sus	
(06025‐532) X W			6	6	Sus	
(03014‐158) X W			1	1	Sus	
(03024‐042) X W			1	1	Sus	
(03124‐791) X W			4	4	Sus	
(03125‐847) X W		1	5	6	Sus	
(06025‐316) X W			1	1	Sus	
(05014‐009) X W	1	1	3	5	Sus	
(05014‐007) X W			1	1	Sus	
(05014‐028) X W			1	1	Sus	
(05014‐029) X W	3		2	5	Sus	
(05014‐124) X W			4	4	Sus	
(05015‐030) X W			9	9	Sus	
Sub‐total	4	7	140	151	Sus	0.92
Total	103	222	424	749		0.95

*Two BC seed families were not tested for disease resistance, and they were presumed to be susceptible based on data from breeding programmes (Hunt, 2004).

Two BC open‐pollinated seed families (BC_#3671 and #3674) and 40 BC *Cr2*‐resistant parental trees were selected to represent three unrelated populations without confirmed phenotypic data. Because no MGR was observed across BC landscapes in all screening tests conducted in the BC breeding programmes (Hunt, 2004), the local seed families (BC_#3671 and #3674) were presumed to be susceptible. Ninety‐three per cent of these samples (28 of 30) were detected with C/C genotype (susceptible). BC *Cr2*‐resistant parental trees were offspring pollinated by bulked pollens from OR *Cr2*‐trees, which were understood to be composed of three genotypes *Cr2/Cr2*,* Cr2/cr2* and *cr2/cr2*. Among them, we detected two trees with A/A and 25 trees with A/C genotypes at the locus snp41490‐1778 (Table 3), providing candidate *Cr2*‐trees for further MGR selection in the BC breeding programmes.

## Discussion

### An integrated genomics‐based strategy for identification of *Cr2* candidates

Based on Mendelian segregation ratios of phenotypes in resistant seed families, four hypothetical genes (*Cr1* to *Cr4*) were proposed as major R genes, with each conferring a species of five‐needle pines (sugar pine, western white pine, south‐western white pine or limber pine) complete resistance against a specific avirulent pathotype of *C. ribicola* (Kinloch and Dupper, 2002; Kinloch *et al*., 1970, 1999; Schoettle *et al*., 2014; Sniezko *et al*., 2008, 2016). Whether these *Cr* genes are the same ortholog is a crucial question in breeding programmes of five‐needle pines (Liu *et al*., 2016a). Molecular characterization of these *Cr* genes is essential for development of MAS tools for breeding applications, which in turn will aid in the development of durable resistance to WPBR (Sniezko *et al*., 2014). In this study, we applied an integrated genomics‐based research strategy for a comprehensive elucidation of the genetic context underlying *Cr2‐*mediated MGR in western white pine.

Our work objectives were achieved by four approaches (Figure 1): (i) the complexity of the *P. monticola* genome was reduced by transcriptome assembly and comparison through RNA‐seq analysis, and SNP loci were targeted to functional candidate genes (Figure 1b); (ii) genic SNP markers were selected for *Cr2* mapping by a G × P association study (Figure 1e) and enriched by a comparative transcriptomics analysis between resistant (*Cr2/cr2*) and susceptible (*cr2/cr2*) seed families of *P. monticola*, or between *P. monticola* and *P. taeda* (Figure 1d); (iii) saturated maps of *Cr2*‐LG were constructed by HRM‐ and TaqMan‐based SNP genotyping using haploid megagametophyte populations of three *Cr2/cr2* seed families, which subsequently led to anchoring *Cr2* on *Pinus* consensus LG‐1 by comparative mapping between *P. monticola* and *P. taeda* (Figure 1f); (iv) genomic regions flanking the *Cr2* locus were determined, resulting in identification of *Cr2* candidates by GO analysis and gene expression profiling (Figure 1g). Our results demonstrated that this integrated genomics strategy is effective for mapping of a genetic locus that controls MGR traits in a conifer species with a large genome, which will help with the discovery and nuclear characterization of other novel R genes against WPBR in five‐needle pines. The TaqMan array snp41490‐1778M was targeted at a NBS‐LRR candidate in the *Cr2* region; and its effectiveness in predicting the presence of *Cr2* in previously uncharacterized populations demonstrated its practical application for use in *P. monticola* breeding and conservation programmes (Table 3; Figure 6). Genic SNP markers and TaqMan arrays can be deployed in future genetic studies of host resistance to WPBR and in searching for additional R alleles in a wider range of *P. monticola* germplasm. *Cr2‐*linked SNP markers, candidate genes and MAS tools provide genomic resources for resistance selection in progenies of MGR‐segregating seed families and WPBR resistance diagnosis in natural stands.

### Independent evolution of five‐needle pine MGR to *C. ribicola*

Genetic mapping of plant traits of interest is an efficient approach widely used for the discovery of DNA variations in molecular plant breeding. Comprehensive understanding of gene alleles contributing to phenotypic variations at the genome level helps plant improvement by genomics‐based practices (Neale *et al*., 2013). In the past two decades, genetic maps have been constructed for *Cr1*,* Cr2* and *Cr4* using various types of DNA markers (Devey *et al*., 1995; Jermstad *et al*., 2011; Liu and Ekramoddoullah, 2007, 2008; Liu *et al*., 2006, 2016a). The recent advances in whole‐genome sequencing and application of NGS have improved genetic mapping of other five‐needle pine species, such as foxtail pine (*P. balfouriana*; Friedline *et al*., 2015). Whole‐genome sequencing identified an NBS‐LRR gene as a candidate of the *P. taeda Fr1* gene against fusiform rust pathogen *C. quercuum* (Berk.) on the *Pinus* consensus LG‐2 (Neale *et al*., 2014; Quesada *et al*., 2013).

*Cr1* and *Cr4* were mapped to the *Pinus* consensus LG‐2 and LG‐8, respectively (Jermstad *et al*., 2011; Liu *et al*., 2016a; Neale *et al*., 2014). In this study, we anchored *Cr2* on the *Pinus* consensus LG‐1. The result provides new evidence supporting our previous hypothesis that five‐needle pine *Cr* genes against WPBR evolved independently before *C. ribicola* invaded into North America (Liu *et al*., 2016a). *C. ribicola vcr1* and *vcr2* pathotypes distinguish themselves by specifically overcoming *Cr1* and *Cr2*, respectively (Kinloch and Dupper, 2002). All this evidence suggests that *Cr‐Avcr* interactions may be different, depending on the five‐needle pine species (Liu *et al*., 2015).

### Does *Cr2* encode an NBS‐LRR protein?

The majority of plant R genes encode NBS‐LRR proteins that initiate plant resistance responses by interacting with pathogen effectors directly or indirectly (McHale *et al*., 2006). Searching for NBS‐LRR genes cosegregated with both qualitative and quantitative R loci has been demonstrated as an effective strategy for the map‐based cloning of R genes (Marone *et al*., 2013). We identified four NBS‐LRR genes in three NBS‐LRR gene clusters and other resistance‐related genes in the *Cr2* region of the *Pinus* consensus LG‐1. No functional R gene has been cloned in gymnosperms. NBS‐LRR genes mapped in this study have strong similarities with the well‐characterized tobacco TMV resistance protein N (Figure S3). All four NBS‐LRR genes mapped within the *Cr2* region are transcriptionally expressed, and two of them were regulated in response to the infection progress in the stem tissues. Similarly, pathogen‐induced transcript expression was previously observed for another *P. monticola* NBS‐LRR gene (Liu and Ekramoddoullah, 2011). These transcript profiles suggest that they may have a role in tree defence in the WPBR pathosystem, even if they may only be R paralogs in the *Cr2* locus.

The majority of R genes tend to be physically organized into gene clusters within *R* loci in plant genomes (Meyers *et al*., 2005). Most R gene clusters are comprised of paralogs with similar NBS‐LRR sequences derived from the same recent common ancestor, indicating that R genes are commonly under rapid evolution in plant genomes via gene duplication and recombination (Yang *et al*., 2013). No SNP markers were cosegregated with *Cr2*. As NBS‐LRR paralogs were clustered into at least three groups in the *Cr2* region, fine dissection of additional paralogs in each cluster will provide more detailed information about genomic organization and evolution of the NBS‐LRR gene family at the *Cr2* locus. In addition to these three mapped NBS‐LRR clusters, we cannot rule out that other NBS‐LRR or RLK genes may be localized in the *Cr2* region. We observed a discontinued collinear relationship at the *Cr2* region compared to the *Pinus* consensus LG‐1, which suggests that a chromosomal rearrangement might have occurred at the *Cr2* region. There is increasing evidence for rearrangements, translocations, gains or losses of DNA segments and copy number variations in all chromosomes among different genotypes of the same species (Swanson‐Wagner *et al*., 2010; Zmienko *et al*., 2014). A further fine mapping of *P. monticola* NBS‐LRR genes and other R gene families (such as RLK) using R gene enrichment sequencing (RenSeq) (Jupe *et al*., 2013), integrated with a genome‐wide association (GWAS) study, would allow fine‐scale dissection of R gene clusters that confer resistance to specific *C. ribicola* avcr races.

Other genes in the *Cr2* region may be involved in host resistance to *C. ribicola*. These include two F‐box genes, both of which showed defence‐responsive expression following *C. ribicola* infection (Figure 5). Contig_4105 encodes a putative F‐box protein with homology to plant FBK. Lineage‐specific FBK genes are unstable with kelch domains that show strong signatures of positive selection, indicating adaptational potential (Schumann *et al*., 2011). In another F‐box gene (contig_11322), the F‐box domain was linked with LRRs, a repeated motif for protein–protein interactions associated with signal transduction networks and other cellular functions (Lechner *et al*., 2006). Plant F‐box genes have been found to control many crucial processes, including pathogen resistance (Lechner *et al*., 2006). An Arabidopsis F‐box protein (CPR1) showed pathogen‐induced expression and functioned as a negative regulator of an R protein (SNC1, an NBS‐LRR protein) likely through protein degradation mediated by a Skp1‐cullin‐F‐box (SCF) complex (Gou *et al*., 2011). Tobacco ACIF1 is an F‐box protein with LRRs with expression up‐regulated after elicitor recognition, and the silencing of its expression suppressed the HR triggered by various elicitors (Van den Burg *et al*., 2008). SCF complex‐mediated stability control of plant R (NBS‐LRR) proteins plays an important role in regulating their protein levels and preventing autoimmunity (Cheng *et al*., 2011).

Contig_2142 encodes a RuvB‐like protein, putatively involved in transcriptional regulation, DNA replication and probably DNA repair (Huen *et al*., 2010). A RuvB‐like protein 1 (AtTIP49a) appears to be a negative regulator of at least some R‐dependent responses (Holt *et al*., 2002). Contig_13194 encodes a bromodomain‐containing protein, which is believed to act as a functional unit for protein–protein interactions (Zeng and Zhou, 2002), directly associating with defence‐related gene regions (Ma *et al*., 2011). Contig_13287 encodes a putative proline‐rich protein (PRP), ubiquitous and implied in the integrity of the cell wall, the structural maintenance of organs, and defence responses to pathogen infection (Fukuoka *et al*., 2009; Kishor *et al*., 2015; Yeom *et al*., 2012).

Contig_573 and contig_3772 encode a putative PDH in the tricarboxylic acid (TCA) cycle and a delta‐fatty acid desaturase, respectively. Both genes may not be *Cr2* candidates, but were up‐regulated following *C. ribicola* infection (Figure 5). PDH was induced upon plant exposure to avirulent pathogens and pathogenic elicitors (Jones *et al*., 2006; Rojas *et al*., 2014; Zulak *et al*., 2009). Plant fatty acid desaturases are responsive to different environmental stresses, including fungal pathogenesis (Kirsch *et al*., 1997). The Arabidopsis desaturase mutation (*ssi2*) confers enhanced resistance to pathogens (Kachroo *et al*., 2001; Mandal *et al*., 2012). These data suggest that the genes mapped in the *Cr2* region may be involved in plant defence response to rust infection.

Although the molecular mechanism by which *Cr2* controls MGR is unclear, identification of multiple genes on the *Cr2*‐LG as functional candidates with defence‐responsive expression opens new approaches to studying the *Cr2‐avcr2* interaction (Liu *et al*., 2015). The recent availability of the *P. taeda* and *P. lambertiana* genome sequence drafts and development of consensus genetic maps for comparative genomic studies among the *Pinus* species (Gonzalez‐Ibeas *et al*., 2016; Neale *et al*., 2014; Stevens *et al*., 2016; Wegrzyn *et al*., 2014; Westbrook *et al*., 2015; Zimin *et al*., 2014) will aid in the molecular characterization of conifer R genes against pathogens (Liu *et al*., 2016a; Neale *et al*., 2014). Sequencing of the genomes of *P. monticola* and other remaining white pine species will improve understanding and developing WPBR resistance in each of them. Our study is an example of an efficient research strategy to identify candidates for R genes in a *Pinus* genome. A further functional analysis of the selected candidates within the *Cr2* cluster will characterize a conifer R gene at the molecular level.

### MAS tools for R gene pyramiding and *Cr2* selection

Breeding programmes of five‐needle pines require practical strategies for the development of elite seed families with durable resistance to WPBR. Before we confirmed that *Cr1‐4* are different R genes, ‘gene pyramiding’ was only a hypothetical strategy in five‐needle pine breeding. Because *Cr1*,* Cr2* and *Cr4* are on different LGs, incorporating multiple R genes into a single seed family for greater resistance durability can be achieved through interspecies hybridization and by transgenic or genome‐editing approaches (Kushalappa *et al*., 2016). However, selection of genotypes with a combination of multiple genes of interest takes a long time in forest breeding practices using conventional approaches and thus the utilization of genomic resources may be able to greatly increase the efficiency of the process (Sniezko *et al*., 2014).

As R genes or QTLs for disease resistance have been mapped in a number of plant species, DNA markers with close linkage have been applied as molecular tools for selection of disease resistance genotypes in crop breeding programmes (Miedaner and Korzun, 2012). Genomics‐based selection of eucalyptus would result in a 50% reduction in time for breeding cycles and a subsequent gain of economic return of 20 times on the investment (Resende *et al*., 2012). Despite promising results from advanced tree genomics studies (Plomion *et al*., 2016; Sniezko *et al*., 2014), practical application of MAS tools in forest breeding has not been reported yet.

In the present study, G × P association analysis identified snp41490‐1778M with *R* ^2^ > 0.9 (*P *< 1.0E−05) (Table 1). This SNP caused an amino acid change (R736/M736), a putative functional mutagenesis in the LRR region of the NBS‐LRR protein. This high level of association between genotype and phenotype makes snp41490‐1778M a robust candidate as a diagnostic marker for *Cr2* selection and for pyramiding *Cr2* with other *Cr* genes, or *Cr2* with partial resistance. This marker would enhance five‐needle pine breeding programmes by increasing the precision and by decreasing the time and cost of the selection process.

The TaqMan tool derived from snp41490‐1778M has high efficiency in detecting resistant genotypes (Table 3), including both homozygous (*Cr2/Cr2*) and heterozygous (*Cr2/cr2*) genotypes due to codominant nature of SNP markers. The first parent trees identified with *Cr2* originated from the Champion Mine area in the Cottage Grove Ranger District of the Umpqua National Forest, OR, USA. This resistant germplasm was planted in British Columbia, Canada, but is not well adapted to the region. The BC breeding programme is currently using conventional breeding to transfer *Cr2* into local seed orchard parents. The local parent trees are highly susceptible to WPBR but have favourable traits, such as fast growth and resistance to root‐rot and needle blight disease. It usually takes 2~3 years for phenotypic confirmation of stem canker development under controlled inoculations in the glasshouse, and much longer in a field test. Using the MAS tools we have developed, we are able to select *Cr2‐*seedlings during seed germination stages. This was confirmed using composite‐resistant (*Cr2/−*) seed families (Table 3), over 500 samples from about 71 full‐sib families (Liu et al., 2014). The other functional SNP markers localized to the NBS‐LRR cluster in the *Cr2* region will be valuable as additional tools to enhance the accuracy of MAS programmes for the improvement of five‐needle pine resistance to WPBR.

## Conclusion

We successfully applied an integrated genomics approach to pinpoint NBS‐LRR and resistance‐related genes as candidates underlying the *Cr2* locus (Figure 1). Genic SNP markers were selected by association analysis and transcriptome comparison. Using these SNP markers, we mapped *Cr2* with saturated resolution on the *Pinus* consensus LG‐1 in three resistant segregating seed families. A nonsynonymous SNP locus of the NBS‐LRR gene in tight linkage with *Cr2* was used to develop a TaqMan array as a diagnostic tool, which opens new prospects for more efficiently breeding of western white pine and other five‐needle pines with more durable resistance against WPBR.

## Materials and methods

### Plant materials and rust inoculation

Megagametophyte samples of three western white pine resistant, segregating (*Cr/cr2*), seed families (#1974, #3566 and #3592) were used as mapping populations. The seedlings from these three maternal trees were confirmed with *Cr2*‐controlled phenotypic segregation in previous inoculation tests, and the *Cr2* locus in these seed families was descended from parents or grandparents from the Champion Mine area in the Cottage Grove Ranger District of the Umpqua National Forest or the Bear Pass planting site on the Willamette National Forest in Oregon (Kinloch *et al*., 1999). Seed families #3566 and #3592 were the same as described previously (Liu and Ekramoddoullah, 2008). Seed family #1974 was grown, inoculated and assessed for resistance‐related traits at the Dorena Genetic Resource Center (DGRC, Cottage Grove, Oregon) as described previously (Danchok *et al*., 2012; Liu *et al*., 2014). In brief, the seeds were sown in June 2010 after 4 months stratification. Megagametophyte (haploid) tissues were individually collected during the first week of seed germination and stored at −20 °C for genomic DNA extraction. Seedlings were grown in a glasshouse and inoculated in September 2010 using basidiospores released from the alternate host *Ribes* (spp.) leaves infected by *avcr2* race. Diseased *Ribes* leaves were collected from geographical areas where no virulent race (*vcr2*) was detected. After infection with *avcr2* race under controlled conditions (basidiospore density ~6000/cm^2^, 100% relative humidity, 18~20 °C) in the inoculation room, seedlings were moved back to the glasshouse. Symptoms of blister rust disease and phenotypic traits were evaluated for each seedling in 2011.

### Selection of SNP markers for *Cr2* mapping

An association study was used as the first method to select genic SNP loci from a *P. monticola* SNP database for construction of a *Cr2* linkage map. Previously, 376 seedlings from a collection of resistant (*Cr2/−*) composite seed families used in the breeding programmes were analysed for 432 SNP loci, and the resistant phenotypes mediated by *Cr2* were evaluated for each individual seedling (Liu *et al*., 2014). These genotypic and phenotypic data were used for a G × P association analysis with a general linear model (GLM) using the software package TASSEL (trait analysis by association, evolution and linkage) version 5.0 (Bradbury *et al*., 2007). *P*‐values for marker effects were adjusted by 100 000 permutations (Churchill and Doerge, 1994).

For the second method to select genic SNP loci for mapping *Cr2*, we performed a comparative transcriptome analysis between resistant (*Cr2/cr2*) and susceptible (*cr2/cr2*) seed families as outlined in a previous report (Liu *et al*., 2014), and 716 *Pinus* highly conserved genes were found to be polymorphic only in *Cr2*‐resistant seedlings but not in *cr2*‐susceptible seedlings. In addition to these genes, other genes identified in the above association analysis, and the DNA sequences of RAPD, AFLP, AFLP‐related RGAs and SCAR markers linked to *Cr2* (Liu and Ekramoddoullah, 2007, 2008; Liu *et al*., 2006), were used as queries in a BLASTn search against the *P. taeda* genome draft (Zimin *et al*., 2014), with special attention to contigs that were mapped on the *P. taeda* LGs (Eckert *et al*., 2009; Neves *et al*., 2014; Westbrook *et al*., 2015). Based on identical hits (*E* value <e−100) in BLASTn analysis and potential physical linkage in *P. taeda* genomic contigs, those highly conserved *Pinus* genes were assessed for their potential linkage to *Cr2* and SNP loci distributed within related genes were then selected from *P. monticola* needle and stem transcriptomes (Liu *et al*., 2013b, 2014). The selected genic SNP loci were screened by HRM‐based SNP genotyping using a subset of megagametophyte samples. GO analysis and gene annotation were performed to annotate putative functions of *Cr2*‐linked genes using the bioinformatics software tool BLAST2GO (Conesa and Götz, 2008).

### SNP genotyping by high‐resolution melting (HRM) analysis

Genomic DNA was extracted from megagametophyte or needle tissues using a Plant DNeasy kit (Qiagen), in accordance with the manufacturer's protocol. SNP loci selected from the above approaches were genotyped using HRM analysis (Gundry *et al*., 2003). HRM primers targeting SNP sites (Table S1) were designed with default criteria using the Primer Express Software (Applied Biosystems, Foster City, CA). PCR mixture consisted of 0.25 μm of forward and reverse primers, 1 × MeltDoctor HRM master mix (Applied Biosystems) and 10 ng of genomic DNA. PCR was run on an ABI 7500 Fast Real‐Time PCR system with programme settings as recommended by the manufacturer. Genotypic data were collected and analysed using HRM Software v2.0 (Applied Biosystems).

To develop MAS tools for *Cr2* selection in breeding programmes, *Cr2*‐linked SNP markers were selected to design custom TaqMan SNP genotyping assays, which contain allele‐specific TaqMan MGB probes with distinct fluorescent dyes and a pair of PCR primers for each specific SNP target (Table S2). Alleles were examined using TaqMan GTXpress master mix and run using a 7500 Fast Real‐Time PCR system (Applied Biosystems) following the manufacturer's instructions. TaqMan Genotyper Software (Applied Biosystems) was used for genotype calling.

Seed families used for genotyping with TaqMan arrays are listed in Table 3 and Table S3. All of them were collected from the OR and BC western white pine breeding programmes. Dr. Rich Hunt (CFS, retired senior research scientist) provided 40 needle samples from BC‐resistant parental trees, and Dr. Valerie Hipkins (USDA‐FS, National Forest Genetics Laboratory) provided 19 genomic DNA samples from OR‐resistant parental trees for testing TaqMan arrays.

### Validation of SNP genotypes by Sanger sequencing

To validate HRM and TaqMan SNP genotyping results, a subset of samples were used for PCR. For Sanger sequencing, amplified genomic DNA fragments targeted at SNP sites were purified using a MinElute PCR Purification Kit (Qiagen) and cloned into the pGEM‐T easy vector (Promega, Madison, Wisconsin, United States). After selection of recombinant plasmids, genomic DNA insert sequences were determined on both strands with an ABI310 DNA sequencer (Applied Biosystems) using a Thermo‐cycle sequence kit (Amersham) with vector primers T7 and SP6. DNA sequence data were assembled and analysed using BLAST, Clustal Omega and ORF finder network services at the National Center for Biotechnology Information (NCBI) and the ExPASy Proteomics Server at the Swiss Institute of Bioinformatics (Geneva, Swiss).

### Linkage group (LG) analysis

The JoinMap software version 3.0 (Van Ooijen and Voorrips, 2001) was used for LG analysis. SNP markers were tested and mapped on the *Cr2‐*LG by haploid segregation analysis as previously described (Liu *et al*., 2006). Each SNP locus was calculated for Mendelian segregation in each seed family by X^2^ (α = 0.05). Markers showing significant (*P* < 0.05) segregation distortions were initially eliminated from the LG construction and were then added later as accessory markers. A logarithm of the odds ratio (LOD) threshold of 6 and a distance threshold of 30 cM were used to define a linkage group (LG). The robustness of the data sets for each LG was confirmed by the grouping module of JoinMap using a LOD threshold of 10. Only megagametophyte samples of the susceptible phenotype were counted because the resistant phenotype could be conferred by *Cr2* from pollen. The Kosambi mapping function was used to calculate genetic distances. The integrated maps were constructed by combining individual maps of three seed families. *P. taeda* consensus maps published previously (Eckert *et al*., 2009; Neves *et al*., 2014; Westbrook *et al*., 2015) were used as references to anchor *Cr2*. Collinearity between LG maps of *P. monticola* and *P. taeda* were determined by identifying the *Pinus* highly conserved genes (*E* values <10 e−100) or orthologs by BLAST analysis and conservation of relative order and location of the mapped genes.

### Gene expression analysis

Illumina RNA‐seq raw reads (SRA run accessions SRR1013833, SRR1013836, SRR1013837, SRR1574690‐1574692 and SRR3273235‐SRR3273237) from previous studies (Liu *et al*.,2013b, 2014) were used to evaluate transcript levels of candidate genes in tissues using CLC genomics workbench v5.5. The *P. monticola* stem transcriptome (Liu *et al*., 2014) was used as a reference to map RNA‐seq reads with parameters: minimum length of putative exons = 50, minimum number of reads = 10, maximum number of mismatches (short reads) = 2, unspecific match limit = 20, minimum exon coverage fraction = 0.2, minimum length fraction (long reads) = 0.9, minimum similarity fraction (long reads) = 0.9. Transcript expression values were measured as reads per kilobase of transcript per million mapped reads (RPKM), but only paired reads were calculated.

Kal's test and *t*‐test were used to estimate statistical significance for differences of transcript levels in the primary needle tissues after *C. ribicola* infection or in the stem tissues between resistant (*Cr2/−*) seedlings (stem canker‐free) and susceptible (*cr2/cr2*) seedlings (stem‐cankered), respectively. In both statistical tests, *P*‐values were corrected by the false discovery rate (FDR).

## Conflict of interest

The authors declare no conflicts of interest.

## Supporting information


**Figure S1** Sanger sequencing for confirmation of SNP loci.

**Figure S2** Comparative mapping of *Pinus* conserved genes between *Pinus monticola Cr2* linkage group (LG) and *P. taeda* consensus LG‐1.

**Figure S3** Alignment analysis of full‐length sequences of *Pinus monticola* contig_41490 and TMV resistance protein N (TMV_N, GenBank Acc: U15605) from tobacco (*Nicotiana glutinosa*).

**Table S1** Primer sequences used for qPCR in genotyping by high‐resolution melting (HRM) analysis.
**Table S2** Primer and probe sequences used for qPCR in SNP genotyping by TaqMan arrays.
**Table S3** Geographical locations of western white pine seed families with open pollination.
