# Supplementary material for: Saturated genic SNP mapping identified functional candidates and selection tools for the Pinus monticola Cr2 locus controlling resistance to white pine blister rust
Source: Plant Biotechnol J. 2017 Mar 17;15(9):1149–62. doi: 10.1111/pbi.12705 (PMC5552481; doi:10.1111/pbi.12705)
Supplement: Supplementary file 1 — Figure S1 Sanger sequencing for confirmation of SNP loci. [file PBI-15-1149-s003.pptx]

## Slide 1
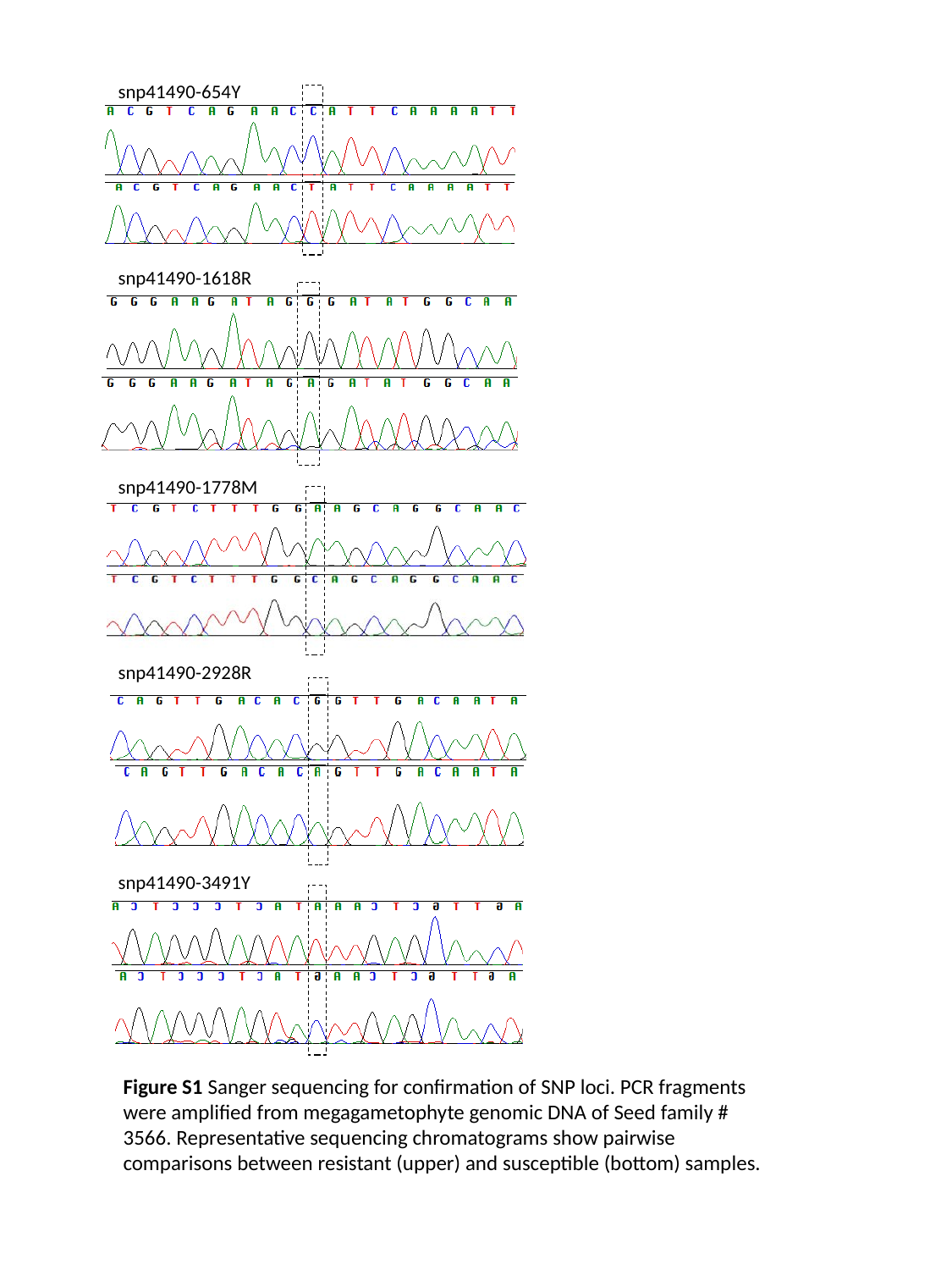

snp41490-654Y
snp41490-1618R
snp41490-1778M
snp41490-2928R
snp41490-3491Y
Figure S1 Sanger sequencing for confirmation of SNP loci. PCR fragments were amplified from megagametophyte genomic DNA of Seed family # 3566. Representative sequencing chromatograms show pairwise comparisons between resistant (upper) and susceptible (bottom) samples.
