# Supplementary material for: Saturated genic SNP mapping identified functional candidates and selection tools for the Pinus monticola Cr2 locus controlling resistance to white pine blister rust
Source: Plant Biotechnol J. 2017 Mar 17;15(9):1149–62. doi: 10.1111/pbi.12705 (PMC5552481; doi:10.1111/pbi.12705)
Supplement: Supplementary file 2 — Figure S2 Comparative mapping of Pinus conserved genes between Pinus monticola Cr2 linkage group (LG) and P. taeda consensus LG‐1. [file PBI-15-1149-s002.pptx]

## Slide 1
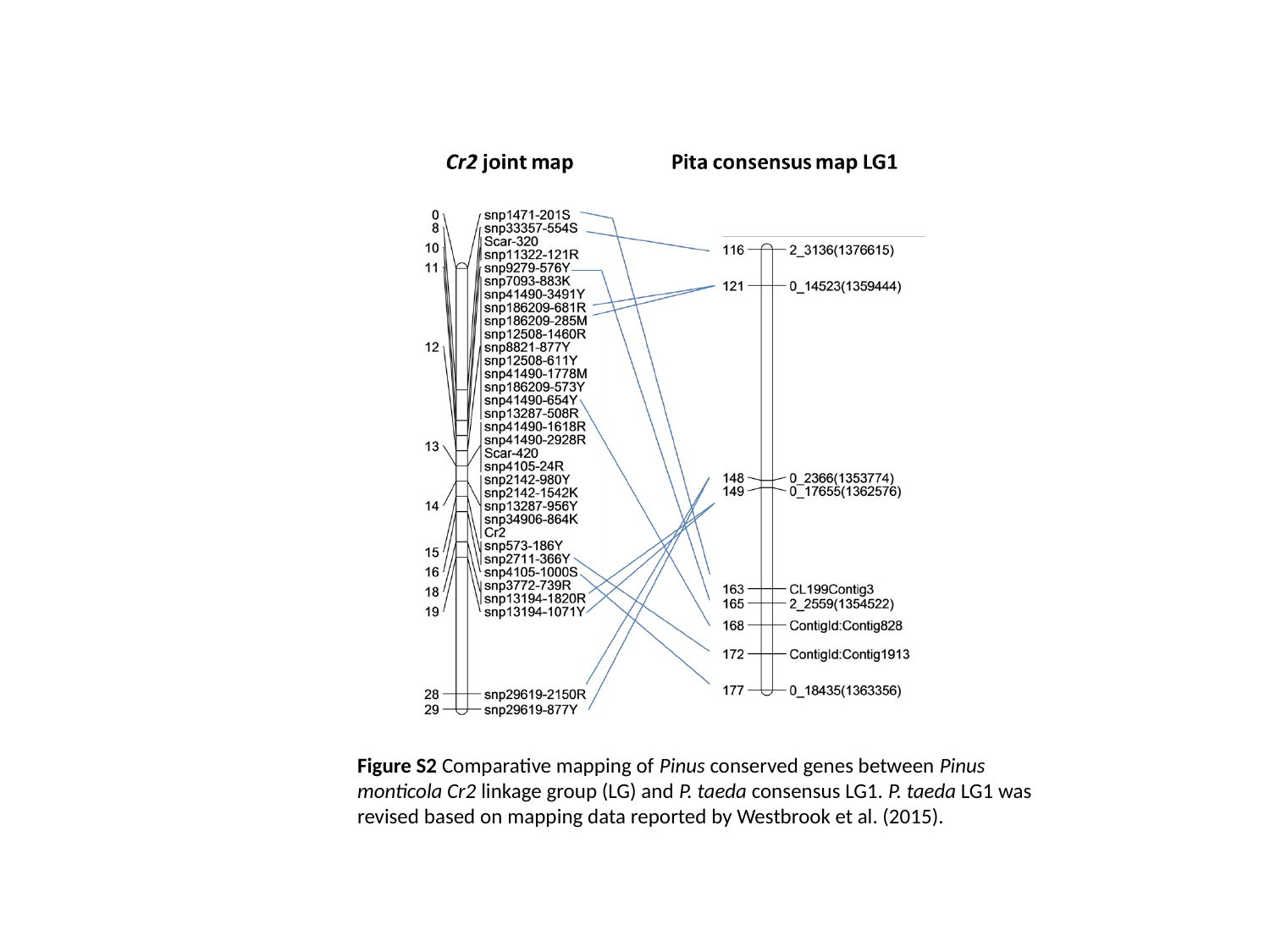

Figure S2 Comparative mapping of Pinus conserved genes between Pinus monticola Cr2 linkage group (LG) and P. taeda consensus LG1. P. taeda LG1 was revised based on mapping data reported by Westbrook et al. (2015).
