# Supplementary figures and images for: Saturated genic SNP mapping identified functional candidates and selection tools for the Pinus monticola Cr2 locus controlling resistance to white pine blister rust
Source: Plant Biotechnol J. 2017 Mar 17;15(9):1149–62. doi: 10.1111/pbi.12705 (PMC5552481; doi:10.1111/pbi.12705)

## Slide 1
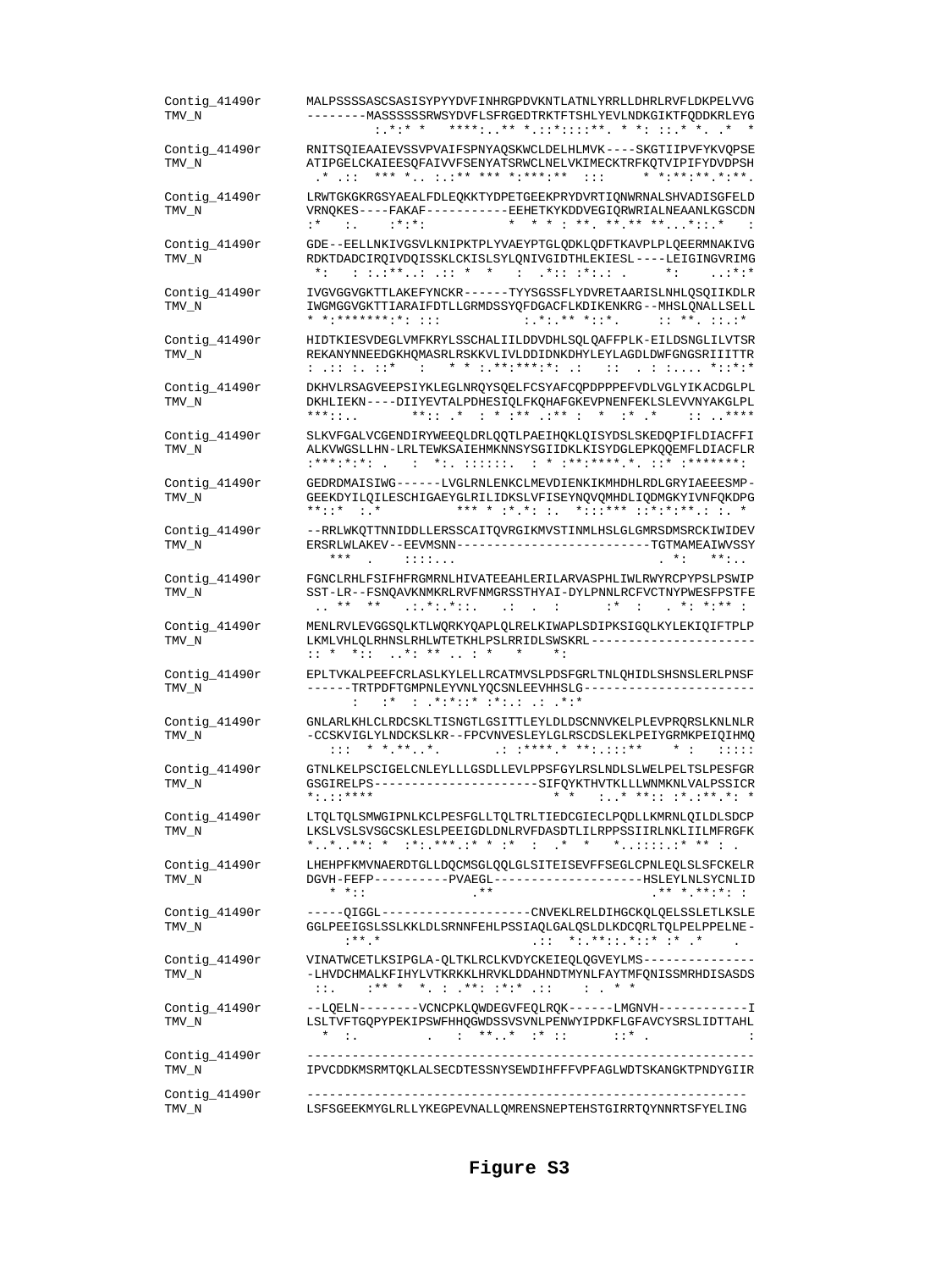

Supplement: Supplementary file 3 — Figure S3 Alignment analysis of full‐length sequences of Pinus monticola contig_41490 and TMV resistance protein N (TMV_N, GenBank Acc: U15605) from tobacco (Nicotiana glutinosa). [file PBI-15-1149-s004.pptx]
